# Supplementary figures and images for: Viral interference between severe acute respiratory syndrome coronavirus 2 and influenza A viruses
Source: PLoS Pathog. 2024 Jul 22;20(7):e1012017. doi: 10.1371/journal.ppat.1012017 (PMC11293641; doi:10.1371/journal.ppat.1012017)

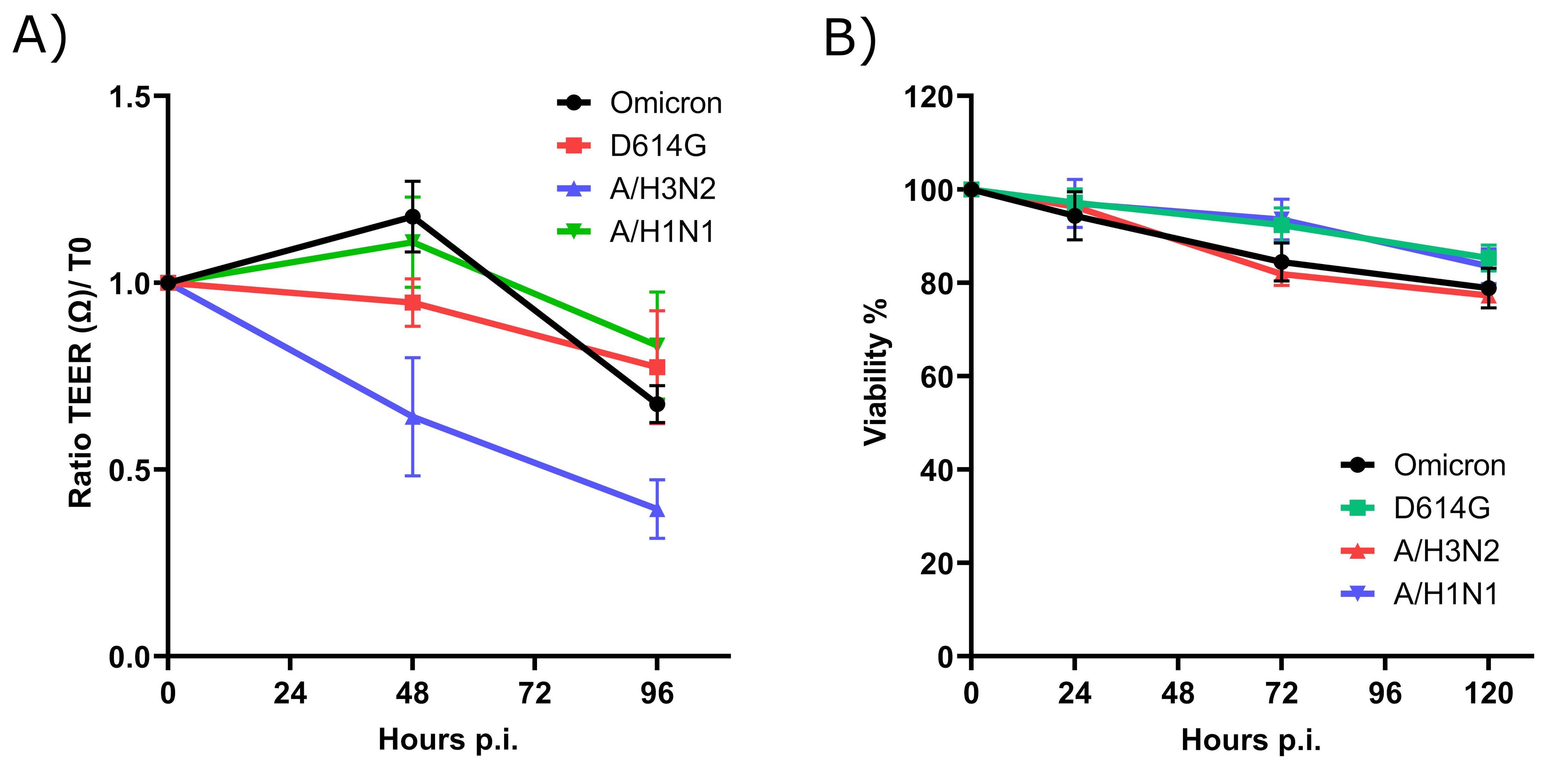

Supplement: S1 Fig — A) Ratio of the trans-epithelial electrical resistance (TEER) over the starting TEER (T0 at day 0) during single infection of HAEs with SARS-CoV-2 (Omicron or D614G) or influenza A (H3N2 or H1N1). B) Percentage of cellular viability over time compared to viability 24 h before infection, determined by a MTS assay. Results represent the mean ± SEM of 3 to 10 replicates from one or two independent experiments. (TIF) [file ppat.1012017.s001.tif]

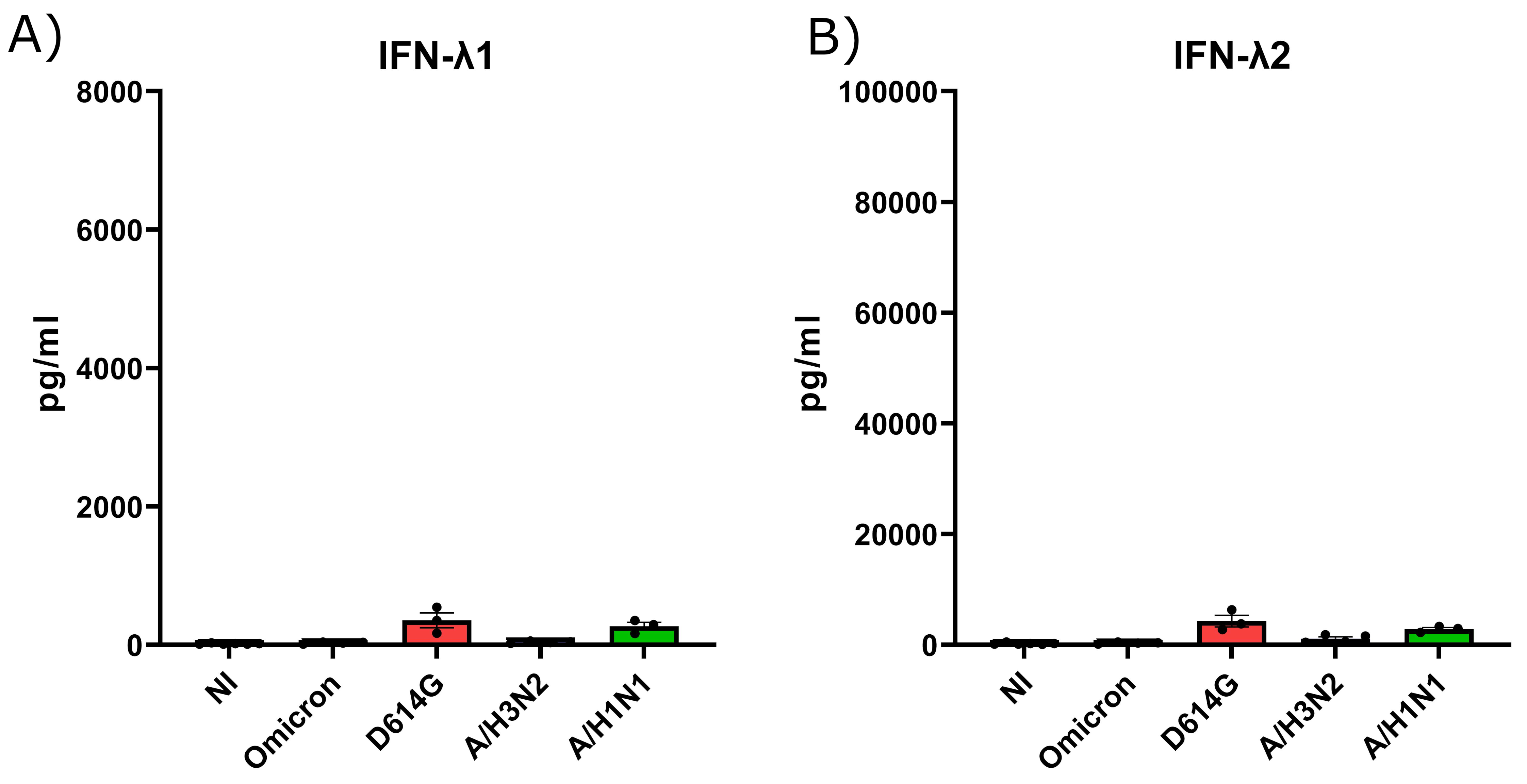

Supplement: S2 Fig — Production of A) IFN-λ1 and B) IFN-λ2 proteins at the basolateral pole of nasal human airway epitheliums (HAEs) after single infections with SARS-CoV-2 (Omicron or D614G) and influenza A (H3N2 or H1N1), at 24 h post-infection. Non-infected HAEs are used as controls (NI). Results are expressed as the mean amount in pg per ml ± SEM of 3 to 4 replicates from one independent experiment. (TIF) [file ppat.1012017.s002.tif]

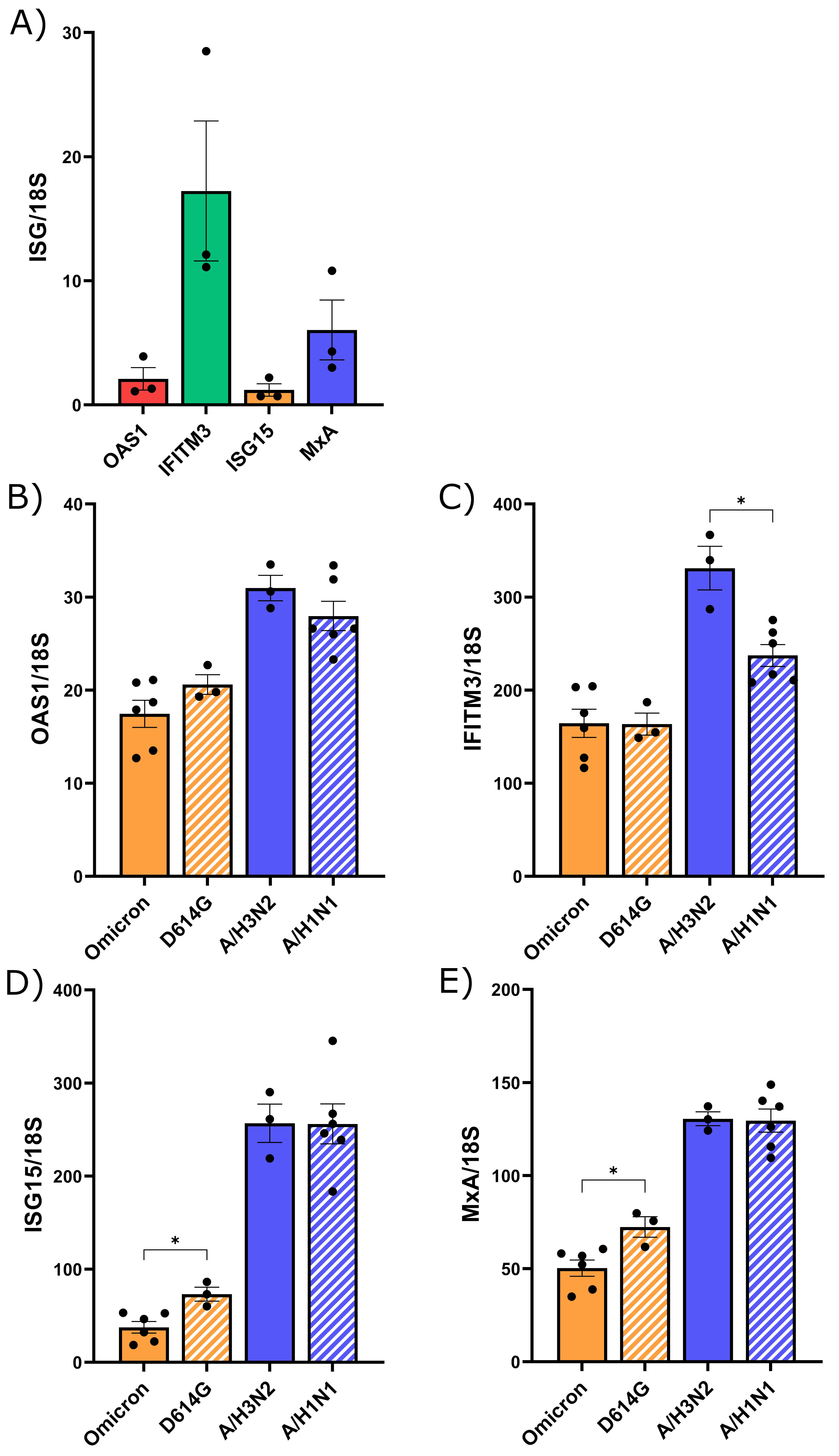

Supplement: S3 Fig — A) Expression of four ISGs (OAS1, IFITM3, ISG15, MxA) in uninfected HAEs. B-D) Comparison of the expression of the different ISGs in HAEs infected with SARS-CoV-2 (Omicron or D614G) or IAV (H3N2 or H1N1) at 120 h p.i. Results are expressed as the mean of the ratio of ISG mRNAs over 18S housekeeping gene (both in copies per μl) ± SEM, calculated using 3 to 6 replicates from one or two independent experiments. *: p ≤ 0.05. (TIF) [file ppat.1012017.s003.tif]

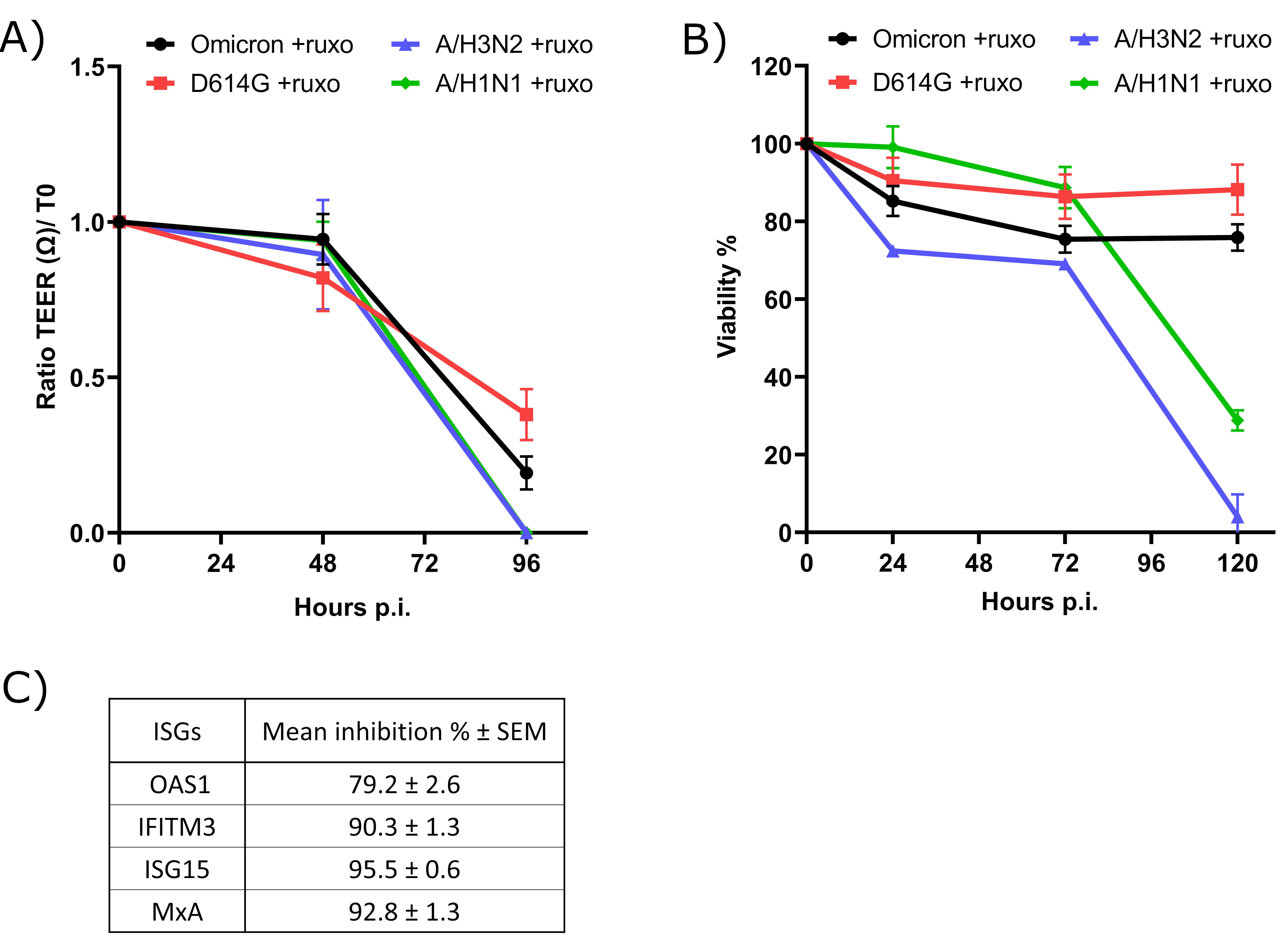

Supplement: S4 Fig — A) Ratio of the trans-epithelial electrical resistance (TEER) over the starting TEER (T0 at day 0) and B) Percentage of cellular viability (determined by a MTS assay) over time compared to viability 24 h before infection and during single infections of HAEs with SARS-CoV-2 (Omicron or D614G) or influenza A (H3N2 or H1N1), in the presence of ruxolitinib (ruxo). C) Inhibition of interferon-stimulated gene (ISG) mRNA expression by ruxolitinib in HAEs infected with both SARS-CoV-2 strains. Results are expressed as the mean inhibition percentage ± SEM of 3 to 10 replicates from two independent experiments. (TIF) [file ppat.1012017.s004.tif]

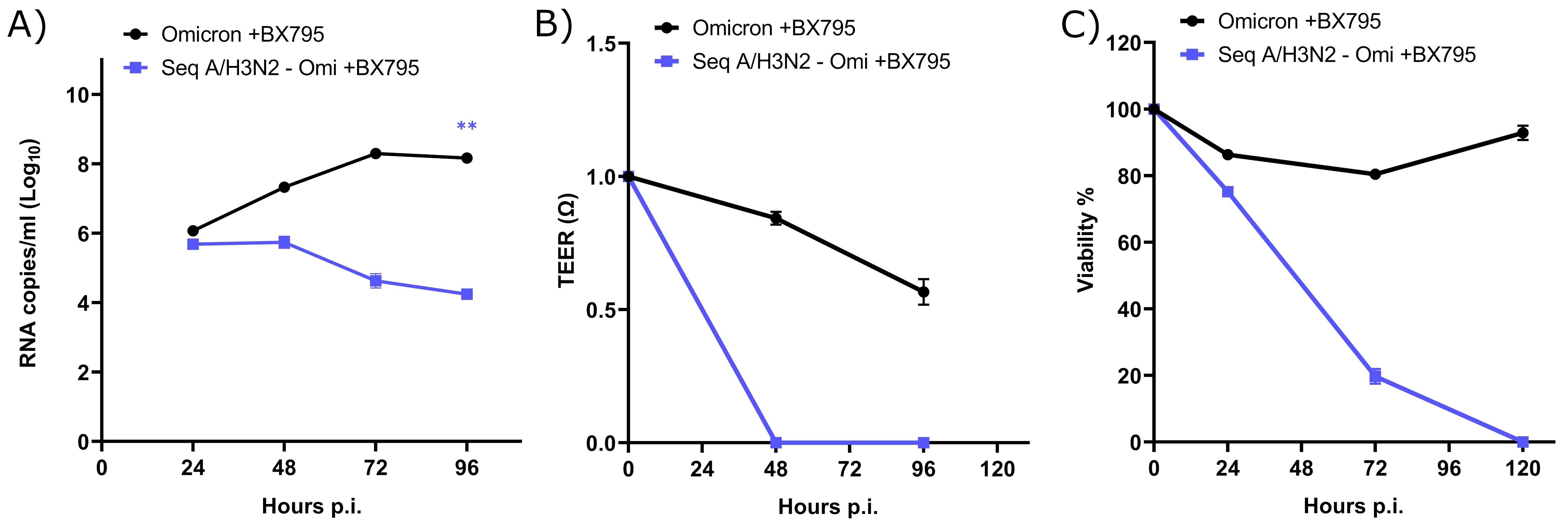

Supplement: S5 Fig — A) Viral RNA loads in nasal human airway epitheliums (HAEs) infected with SARS-CoV-2 Omicron alone or in sequential coinfections (seq) 24 h after A/H3N2, in the presence of BX795. Results are expressed as the mean of the Log10 of viral RNA copies per ml ± SEM of 3 replicates from one experiment. **: p ≤ 0.01. B) Ratio of the trans-epithelial electrical resistance (TEER) over the starting TEER (T0 at day 0) and C) Percentage of cellular viability (determined by a MTS assay) over time in HAEs infected with Omicron alone or sequentially with A/H3N2 and Omicron, compared to viability 24 h before infection, in the presence of BX795. Results represent the mean ± SEM of 3 replicates from one experiment. (TIF) [file ppat.1012017.s005.tif]
