# Supplementary material for: Viral interference between severe acute respiratory syndrome coronavirus 2 and influenza A viruses
Source: PLoS Pathog. 2024 Jul 22;20(7):e1012017. doi: 10.1371/journal.ppat.1012017 (PMC11293641; doi:10.1371/journal.ppat.1012017)
Supplement: S1 Table — (DOCX) [file ppat.1012017.s006.docx]

**S1 Table.** **Sequences of primers and probes used for quantification of interferon-stimulated genes and a housekeeping gene by ddPCR.**

|  |  | Sequence | Reference |
| --- | --- | --- | --- |
| **OAS1** | Forward | 5’-GCA AAC AGG TCT GGG AGG-3’ | This work |
|  | Reverse | 5’-GTC AAT GGC ATG GTT GAT TTG C-3’ | This work |
|  | Probe | 5’-CAG TTC TGT TGC CAC TCT CTC TCC TG-3’ | This work |
| **IFITM3** | Forward | 5’-ATC GTC ATC CCA GTG CTG AT-3’ | (Cheemarla et al., 2021)^1^ |
|  | Reverse | 5’-ATG GAA GTT GGA GTA CGT GG-3’ | (Cheemarla et al., 2021)^1^ |
|  | Probe | 5’-CAG GAG GCA TCA CTG AGG CCA G-3’ | This work |
| **ISG15** | Forward | 5’-TGG ACA AAT GCG ACG AAC C-3’ | This work |
|  | Reverse | 5’-GGT CAG CCA GAA CAG GTC-3’ | This work |
|  | Probe | 5’-CTG GTG AGG AAT AAC AAG GGC CGC-3’ | This work |
| **MxA** | Forward | 5’-GTC AGT TAC CAG GAC TAC GA-3’ | This work |
|  | Reverse | 5’- ATC TCC AGG GTG ATT AGC TC-3’ | This work |
|  | Probe | 5’-TTG AGA TTT CGG ATG CTT CAG AGG TAG-3’ | This work |
| **18S** | Forward | 5’-GGA TGC GTG CAT TTA TCA G-3’ | This work |
|  | Reverse | 5’-AGT TGA TAG GGC AGA CGT TC-3’ | This work |

^1^ Cheemarla NR, Watkins TA, Mihaylova VT, Wang B, Zhao D, Wang G, et al. Dynamic innate immune response determines susceptibility to SARS-CoV-2 infection and early replication kinetics. J Exp Med. 2021;218(8). Epub 20210615. doi: 10.1084/jem.20210583. PubMed PMID: 34128960.
